# A DNA extraction protocol for improved DNA yield from individual mosquitoes

**DOI:** 10.12688/f1000research.7413.1

**Published:** 2015-11-20

**Authors:** Catelyn C. Nieman, Youki Yamasaki, Travis C. Collier, Yoosook Lee

**Affiliations:** 1 Vector Genetics Laboratory, Department of Pathology, Microbiology, and Immunology, University of California, Davis, CA, USA

**Keywords:** DNA extraction, mosquito, molecular biology

## Abstract

Typical DNA extraction protocols from commercially available kits provide an adequate amount of DNA from a single individual mosquito sufficient for PCR-based assays. However, next-generation sequencing applications and high-throughput SNP genotyping assays exposed the limitation of DNA quantity one usually gets from a single individual mosquito. Whole genome amplification could alleviate the issue but it also creates bias in genome representation. While trying to find alternative DNA extraction protocols for improved DNA yield, we found that a combination of the tissue lysis protocol from Life Technologies and the DNA extraction protocol from Qiagen yielded a higher DNA amount than the protocol using the Qiagen or Life Technologies kit only. We have not rigorously tested all the possible combinations of extraction protocols; we also only tested this on mosquito samples. Therefore, our finding should be noted as a suggestion for improving people’s own DNA extraction protocols and not as an advertisement of a commercially available product.

## Introduction

DNA extraction for *Anopheles* mosquitoes is typically done using commercially available products ( Brown *et al.*, 2011; Demirci *et al.*, 2012; Horton *et al.*, 2010; Main *et al.*, 2015; Norris *et al.*, 2015; Weetman *et al.*, 2012). They work well enough to provide a sufficient amount of DNA for PCR-based assays from a single mosquito. However, next-generation sequencing applications and high-throughput SNP genotyping assays exposed the limitation of DNA quantity from a single mosquito using typical extraction protocols ( Marsden *et al.*, 2011). Whole genome amplification could alleviate the issue but it also creates bias in genome representation (see Table 1 and results section below).

**Table 1.** Whole genome sequencing quality comparison between original and whole-genome-amplified (WGA) DNA. Increase or decrease in comparison with sequence from original DNA is marked in up or down arrows in parentheses.

				Insert size	Mean coverage							
Sample	DNA type	Total reads	% Unmapped	Median	Whole	X	2L	2R	3L	3R	UNKN	chrM
*A. gambia* 1	WGA	63,903,121 (↑)	1.22% (↓)	318 (↑)	27.02 (↑)	26.5	20.86	47.8	18.6	21.38	15.39	57.23 (↓)
	original	17,720,693	1.76%	185	7.19	8.15	7.29	6.71	6.9	6.91	8.12	897.11
*A. gambia* 2	WGA	42,663,199 (↑)	17.46% (↓)	92 (↓)	11.06 (↑)	21.4	8.21	6.28	11.6	7.87	13.64	38.48 (↓)
	original	1,893,678	33.37%	141	0.38	0.43	0.39	0.37	0.37	0.36	0.36	214.62
*A. gambia* 3	WGA	34,384,169 (↑)	76.98% (↑)	462 (↑)	3.75 (↑)	4.48	3.79	3.87	3.19	3.4	2.06	10.61 (↓)
	original	11,877,569	21.71%	261	3.30	3.62	3.32	3.23	3.16	3.18	2.77	132.53
*A. arabiensis* 1	WGA	30,334,217 (↑)	8.07% (↑)	464 (↓)	13.47 (↑)	16.93	12.99	15	10.4	12.11	7.03	5.7 (↓)
	original	14,357,720	4.21%	489	6.20	9.8	5.4	5.2	5.3	5.4	10.2	11.9

While trying to find alternative DNA extraction protocols for improved DNA yield but without compromising the automation option, we found that a combination of tissue lysis protocol from Life Technologies and DNA extraction protocol from Qiagen yielded higher DNA amount than the protocol using Qiagen or Life Technologies kit only.

## Method

### Samples

Adult mosquitoes from a single generation of Pimperena and Mopti-NIH colonies obtained from Malaria Research and Reference Reagent Resources Center (Manassas, VA) were used for this study. Prior to extraction, the whole adult mosquito samples are individually preserved either by freezing at -20°C or storing in 80% ethanol; for the latter case, samples are rehydrated in water for 1 hour prior to DNA extraction.

### Tissue lysis using Life Technologies MagMAX protocol

We used a protocol adapted from the manufacturer’s mouse tail protocol and Whatman FTA card protocol. For each sample contained in a 1.5mL tube, 8µL or 2µL of proteinase K (Life Technologies, 100mg/mL concentration) were added with 92µL or 98µL PK buffer (Life Technologies), respectively. Three specimens were processed without physical disruption. A 3mm diameter steel bead was added to each of the rest of samples and homogenized using Qiagen Tissulyser (Qiagen, Valencia, CA) for 30sec at 30Hz. Mosquito tissue in PK buffer and proteinase K solution were incubated for 2 hours at 56°C. After incubation, 100µL of DNA lysis buffer was added to each tube. Each tube was vortexed briefly (<10s) and centrifuged at 15,000rpm using an Eppendorf microcentrifuge. This created white precipitate, which was mixed by pipetting up and down several times before transferring to a 2.0mL deep well plate for DNA extraction. 90µL of lysate was used for DNA extraction using the Biosprint 96 instrument. The other 90µL of lysate from the same sample was used for DNA extraction using the MagMAX™ Express-96 Magnetic Particle Processor.

### Tissue lysis using Qiagen BioSprint protocol

We used a protocol adapted from the manufacturer’s tissue extraction protocol. For each sample contained in a 1.5mL tube, 10µL or 40 µL of proteinase K (Qiagen, 20mg/mL concentration) were added with 90µL or 60µL ATL buffer (Qiagen, Vallencia, CA), respectively. A 3mm diameter steel bead was added to each of the remaining samples and homogenized using Qiagen Tissulyser (Qiagen, Valencia, CA) for 30sec at 30Hz. Mosquito tissue in ATL buffer and proteinase K solution were incubated for 2 hours at 56°C. Each tube was centrifuged briefly after incubation. 90µL of lysate was used for DNA extraction using the Biosprint 96 instrument. The other 90µL of lysate from the same sample was used for DNA extraction using the MagMAX instrument.

### DNA extraction using Life Technologies MagMAX protocol

As noted earlier, we used a protocol adapted from the manufacturer’s mouse tail protocol and Whatman FTA card protocol. 120µL of 100% isopropanol was added to each lysate. The plate containing lysate and isopropanol was gently mixed (220rpm) using a shaker for 3 minutes. 20µL of DNA binding bead mix (16µL binding beads and 4µL of PCR-grade water) was added to each sample and shook for 3 minutes at 220rpm. We used the “4412021 DW Blood” protocol on the MagMAX instrument, which washes lysate once with wash buffer 1 and twice with wash buffer 2 (Life Technologies). Initial heated elution volume was 75µL (elution buffer 1) and then when prompted, 75µL of elution buffer 2 was added to complete the DNA elution step.

### DNA extraction using Qiagen Biosprint protocol

100µL of 100% isopropanol, 100µL of AL buffer (Qiagen) and 15µL of MagAttract Suspension (Qiagen) was added to each lysate. We used the “BS96 DNA Tissue” protocol on the BioSprint 96 instrument, which washes lysate twice with AW1 buffer, twice with AW2 buffer (Qiagen), and once with water with added tween 20 (Sigma) at a final concentration of 0.02%. The DNA was eluted in 150µL AE buffer (Qiagen).

### DNA quantification & analysis

DNA yield was measured using a Qubit high sensitivity, double stranded DNA kit (Life Technologies), using 1µL of input DNA. R statistics software version 3.0.0 was used to calculate mean and standard deviation and to perform Wilcoxon rank sum test with α of 0.05 after multiple comparison.

### Whole genome sequencing

5µL of original input DNA was used to amplify the whole genome using Qiagen Repli-g kit. We followed the manufacturer’s protocol. We followed the library protocol provided in Norris *et al.* (2015). Genomic DNA libraries were sequenced using Illumina HiSeq2500 platform with paired-end 150 bp reads at the QB3 Vincent J Coates Genomics Sequencing Laboratory at UC Berkeley. Adaptor sequences and poor quality sequences were trimmed from the raw Illumina fastq files using the Trimmomatic software version 0.30 ( Bolger *et al.*, 2014) using default options. Reads were aligned to the *A. gambiae* reference genome AgamP3 ( Giraldo-Calderon *et al.*, 2015) using BWA-MEM version 0.7.5 ( Li, 2013).

## Results & Discussion

Raw data for ‘A DNA extraction protocol for improved DNA yield from individual mosquitoes’ http://dx.doi.org/10.5256/f1000research.7413.d107517
Sample: An artibtrary sample identification code; Group: Particular protocol id listed in Table 3 in the main paper; Read 1: DNA concentration reading in ng/uL using Qubit instrument; Read 2: DNA concentration reading in ng/uL using Qubit instrument using different standard curve; Read 3: DNA concentration reading in ng/uL using Qubit instrument using different standard curve; DNA concentration: Average DNA concentration of Read 1–3 as consensus DNA quantification; Overall mean: The mean of DNA concentrations for each group; std: The standard deviation of DNA concentrations for each group; %(>0.375ng/uL): The percentage of samples yielded greater than 0.375ng/uL DNA. Click here for additional data file. Copyright: © 2015 Nieman CC et al. 2015 Data associated with the article are available under the terms of the Creative Commons Zero "No rights reserved" data waiver (CC0 1.0 Public domain dedication).

In our attempt to sequence whole genomes from field-collected individual mosquitoes, about 50% of specimens failed to pass the DNA quantity required (>30ng) for whole genome sequencing. These DNA samples were extracted using our established DNA extraction protocols using Qiagen kits and instruments ( Lee *et al.*, 2009; Main *et al.*, 2015; Marsden *et al.*, 2011; Norris *et al.*, 2015; Slotman *et al.*, 2006). The requirement of high genomic DNA content is not new. In the past, people have circumvented the problem by conducting whole genome amplification ( Lee *et al.*, 2013; Marsden *et al.*, 2011; Weetman *et al.*, 2012). We sequenced the whole genomes using original DNA in parallel with whole-genome amplified DNA to test if we can use whole genome amplification to bypass the DNA quantity issue.

Whole genome amplified DNA provided a higher number of reads than the original DNA with less DNA input ( Table 1). However, comparison revealed that the particular whole genome amplification kit we used is not suitable for retrieving certain sections of genomes such as mitochondrial genome. This is indicated in the lower depth of coverage in mitochondrial genome in whole genome amplified material while the rest of chromosomes had higher depths relative to the library from original DNA. More importantly, the sequence generated from whole genome amplified samples produced number of inconsistent genotype calls ( Table 2). This inconsistency became more apparent in mitochondrial sequences where heterozygous calls were produced where the genome sequence from the same original DNA had no such calls. These biases are likely introduced by the random primers used in the whole genome amplification kit. This result prompted us to pursue developing better DNA extraction protocols to improve DNA yield in an automated setup.

**Table 2.** Genotype call comparison for selected loci. Chr stands for chromosome, locus for genomic coordinates, A1 for allele 1, A2 for allele 2, angle for arctangent value from two variable (=atan2(A1,A2)), and GT for genotype calls based on angle value. If arctangent value is less than 0.25, it is considered as A1/A1 homozygote. If value is higher than 1.25, genotypes are called as A2/A2. Otherwise, genotoypes are called as heterozygote (A1/A2).

			Original DNA				WGA				
Sample	Chr	Locus	A1 depth	A2 depth	ANGLE	GT	A1 depth	A2 depth	ANGLE	GT	Same?
*A. gambiae 1*	Mt	3164	189	0	0	A1/A1	9	3	0.3218	A1/A1	yes
*A. gambiae 1*	Mt	3211	168	0	0	A1/A1	17	4	0.2311	A1/A1	yes
*A. gambiae 1*	2R	19625228	2	0	0	A1/A1	56	0	0	A1/A1	yes
*A. gambiae 1*	2R	19625240	3	0	0	A1/A1	1	59	1.5538	A2/A2	NO
*A. gambiae 1*	2R	19625389	0	0	-	no call	0	73	1.5708	A2/A2	NO
*A. gambiae 1*	2R	19650738	2	0	0	A1/A1	1	10	1.47118	A2/A2	NO
*A. gambiae 1*	2R	19728934	1	1	0.7854	A1/A2	0	7	1.5708	A2/A2	NO
*A. gambiae 1*	2R	19738843	3	0	0	A1/A1	3	10	1.27938	A2/A2	NO
*A. gambiae 1*	X	23752288	2	1	0.4636	A1/A2	2	2	0.7854	A1/A2	yes
*A. gambiae 1*	X	23756047	3	0	0	A1/A1	2	1	0.4636	A1/A2	NO
*A. gambiae 1*	X	23779162	0	4	1.5708	A2/A2	0	0	-	no call	NO
*A. gambiae 2*	Mt	8276	714	1	0.0014	A1/A1	7	8	0.8520	A1/A2	NO
*A. gambiae 2*	Mt	8840	3	774	1.5669	A2/A2	0	6	1.5708	A2/A2	yes
*A. gambiae 2*	Mt	8927	22	817	1.5439	A2/A2	4	6	0.9828	A1/A2	NO
*A. gambiae 2*	Mt	9098	559	0	0	A1/A1	8	2	0.2450	A1/A1	yes
*A. gambiae 2*	2R	19850861	14	5	0.3430	A1/A2	4	2	0.4636	A1/A2	yes
*A. gambiae 2*	2R	19850867	17	1	0.0588	A1/A1	5	0	0	A1/A1	yes
*A. gambiae 2*	2R	19851283	9	8	0.7266	A1/A2	4	2	0.4636	A1/A2	yes
*A. gambiae 2*	X	22010492	6	3	0.4636	A1/A2	8	1	0.1244	A1/A1	NO
*A. gambiae 2*	X	22011555	9	6	0.5880	A1/A2	4	63	1.5074	A2/A2	NO
*A. gambiae 2*	X	22013224	10	3	0.2915	A1/A1	60	1	0.0167	A1/A1	yes

We found that the Life Technologies tissue lysis and extraction protocol ( Table 3, line 4, in purple) was highly consistent in its DNA yield. A combination of the Life Technologies tissue lysis with the Qiagen BioSprint DNA extraction protocol ( Table 3, line 3, in green) gave the highest average DNA yield (Wilcoxon rank sum test P-value=0.0031). The amount of magnetic beads added to tissue lysate had little effect on DNA yield. The amount of proteinase K (2µL vs 8µL) also showed little difference in DNA yield. Chemical lysis alone, without physical disruption, was not sufficient to produce consistency in DNA yield ( Table 3, lines 5 and 6).

**Table 3.** DNA yield for different combinations of lysis and DNA extraction protocols. Group 1 is the standard protocol used at the Vector Genetics Laboratory over ten years.

Group	Lysis	Physical disruption	DNA extraction	Mean conc. (ng/µL)	SD	% (>0.375ng/µL)
1	Qiagen proteinase K + ATL	Yes	Qiagen Biosprint	0.63	0.63	50.0%
2	Qiagen proteinase K + ATL	Yes	LifeTech MagMAX	0.74	0.20	83.3%
3	LifeTech proteinase K + PK + lysis buffer	Yes	Qiagen Biosprint	1.33	0.80	92.9%
4	LifeTech proteinase K + PK + lysis buffer	Yes	LifeTech MagMAX	0.84	0.07	100.0%
5	LifeTech proteinase K + PK + lysis buffer	No	Qiagen Biosprint	0.88	0.55	66.7%
6	LifeTech proteinase K + PK + lysis buffer	No	LifeTech MagMAX	0.56	0.33	66.7%

For a typical PCR-based assay, DNA quantity of 0.25–1.8 ng/µL in 200µL volume is sufficient. However, genomic approaches such as whole genome DNA library construction for next generation sequencing demand as little as 30ng of DNA. In our typical Qiagen BioSprint DNA extraction protocol, roughly 50% of DNA samples failed to yield 0.375ng/µL ( Table 3, line 1, in blue), which leaves ~50µL of DNA for future study and allows for only a single trial of whole genome library construction. This constraint became a significant hindrance in our research involving whole genome sequencing from an individual mosquito.

This improved DNA extraction protocol will increase the chance of library construction from a single individual. Our observation is limited to trying out commercially available automated DNA extraction protocols and we do not have sufficient expertise on why certain protocols worked better or worse than others. As genomic approaches are more readily available to researchers, this improved DNA extraction protocol will facilitate such approaches that demand high-quantity DNA input from limited source material.

We have not rigorously tested all the possible combinations of extraction protocols. We only tested this on mosquito samples, and we only explored high-throughput automated DNA extraction protocols as we typically handle hundreds of mosquito samples at a time for population genetics studies. Therefore, our findings should be noted as suggestion for improving people’s own DNA extraction protocols and not as an advertisement of a commercially available product.

## Data availability

The data referenced by this article are under copyright with the following copyright statement: Copyright: © 2015 Nieman CC et al.

Data associated with the article are available under the terms of the Creative Commons Zero "No rights reserved" data waiver (CC0 1.0 Public domain dedication). 

*F1000Research*: Dataset 1. Raw data for ‘A DNA extraction protocol for improved DNA yield from individual mosquitoes’, 10.5256/f1000research.7413.d107517 ( Nieman *et al.*, 2015).
