# Supplementary material for: A DNA extraction protocol for improved DNA yield from individual mosquitoes
Source: F1000Res. 2015 Nov 20;4:1314. [Version 1] doi: 10.12688/f1000research.7413.1 (PMC4743141; doi:10.12688/f1000research.7413.1)
Supplement: Peer review history

# Peer review: Referee response for version 1

**DOI:** 10.5256/f1000research.7988.r11970

**Published:** 2016-02-10

**Authors:** Karla Saavedra-Rodriguez

**Affiliations:** 1 Department of Microbiology, Immunology and Pathology, Colorado State University, Fort Collins, CO, USA

The manuscript provides very useful data for researchers aiming to analyze whole mosquito genomes; especially when sequencing is still expensive and having a reliable DNA extraction protocol is essential. The abstract, justification, protocols and results are very well described.

Some comments:

- Did the preservation of individual mosquitoes – 80% alcohol or -20°C–  affect the DNA yield? I am not sure if this was an additional variable. Please clarify.
-  Mention the number of specimens you used for each combination of lysis and extraction methods.
- Table 1: Do ‘X, 2L, 2R, 3L, ..chrM’ columns correspond to mean coverage’s? Please add a brief description of these terms in the table legend.
- Discussion: the statement ‘the amount of magnetic beads…had little effect on DNA yield’. This variable was not mentioned in the methods section. Please add, mention specific bead amounts tested, sample size and statistical test.
- Table 2: Please mention in this table (or table 1) what ‘Mt, 2R….X’ stands for. Are ‘Mt and chM’ mitochondrial DNA?
- Table 2: State the difference between *A. gambiae* 1 and *A. gambiae *2 groups.
- Table 2: There are significant differences between the genotype-calls in the WGA and original DNA. Would you suggest having a third method to validate your genotypes?  Maybe regular DNA sequencing. I think this could add to your discussion.
- Table 3: Add a column with your sample sizes.

I have read this submission. I believe that I have an appropriate level of expertise to confirm that it is of an acceptable scientific standard.

# Peer review: Referee response for version 1

**DOI:** 10.5256/f1000research.7988.r11972

**Published:** 2016-02-01

**Authors:** Zainulabeuddin Syed, Paul Hickner

**Affiliations:** 1 Department of Biological Sciences, University of Notre Dame, Notre Dame, IN, USA; 2 Department of Biological Sciences, University of Notre Dame, South Bend, IN, USA

This m/s by Niema *et al*. presents some useful information for researchers who routinely struggle with reliable protocols to extract high quality DNA, esp. of great quality needed for sequencing. Library construction for next-generation sequencing commonly requires at least 30 ng of DNA. This can present a challenge for researchers that work with small insects and/or who are very limited based on the tissues they use for library construction. DNA yield is particularly important when studying genetic variation among individuals. The authors assessed several common methods for the extraction of DNA from single *Anopheles* mosquitoes. The manuscript is well written and the methods are appropriate.

**Comments:**

No “non-commercial” methods were included in the analyses, nor were they discussed. For example, a standard CTAB protocol gets excellent yields of good quality DNA. Perhaps CTAB does not produce DNA that is appropriate for library prep or does not work well in an automated system. It would be really helpful if authors can address and elaborate on this issue

Were the sizes of the individuals used in the study the same? For example, the mass of males is often much less than females, and rearing conditions can influence size to a great extent.   

Please correct a few spelling errors.

We have read this submission. We believe that we have an appropriate level of expertise to confirm that it is of an acceptable scientific standard.

# Peer review: Referee response for version 1

**DOI:** 10.5256/f1000research.7988.r11616

**Published:** 2015-12-17

**Authors:** Beniamino Caputo, Verena Pichler

**Affiliations:** 1 Department of Public Health and Infectious Diseases, Sapienza University of Rome, Rome, Italy; 2 Department of Public Health & Infectious Diseases, Sapienza University of Rome, Rome, Italy

The article is an interesting update on extraction protocols and highlights the importance of testing different methods when extracting DNA for a wide range of downstream applications. The article’s title is sound and describes well the observations made, the same is true for the abstract which provides an adequate summary of the article. Also the aim of the article and the study design are well described and allow to reproduce the different methods tested. Results and discussions appear to be sound and coherent with the offered data.

There are only few minor corrections to make:

In Methods: 

- please correct TissuLyser in TissueLyser
- specify the number of specimens analyzed

 

In Results and Discussion:

- in the second paragraph it might be noteworthy that genotype call errors do not appear only when considering the mitochondrial DNA, but, at least in *A.gambiae* 1 listed in Table 2, many errors , such as opposed genotype calls can be observed also for autosomal sequences

 

Table 1:

- it could be useful to add some information in the caption such as the meaning of ChrM ( I suppose it stands for mitochondrial DNA?) and units of measurements for the given data
- please correct *A.gambia* in *A.gambiae *(1st column)

We have read this submission. We believe that we have an appropriate level of expertise to confirm that it is of an acceptable scientific standard.
